# Supplementary material for: Ultra-narrowband and rainbow-free mid-infrared thermal emitters enabled by a flat band design in distorted photonic lattices
Source: Nat Commun. 2024 May 13;15:4019. doi: 10.1038/s41467-024-48499-4 (PMC11091152; doi:10.1038/s41467-024-48499-4)
Supplement: Supplementary file 1 — Supplementary Information [file 41467_2024_48499_MOESM1_ESM.pdf]

Supplementary Information for:

Ultra-narrowband and rainbow-free mid-infrared thermal emitters enabled by a flat band design in distorted photonic lattices

Kaili Sun[1], Yangjian Cai[1], Lujun Huang[2,*] and Zhanghua Han[1,*]

[1] *Shandong Provincial Key Laboratory of Optics and Photonic Devices, Center of Light Manipulation and Applications, School of Physics and Electronics, Shandong Normal University, Jinan 250358, China*

[2]*School of Physics and Electronic Science, East China Normal University, Shanghai, 200241, China*

**Corresponding author: ljhuang@phy.ecnu.edu.cn, zhan@sdnu.edu.cn*

I. Material parameters for the simulations

The materials used in this paper includes Au, Al_2O_3, Ge.

Au: In numerical simulation, the dielectric constant of Au can be characterized by means of a Drude model as follows[1,2]:

$$\varepsilon_{Au} = \varepsilon_{\infty} - \frac{\omega_p^2}{\omega^2 + i\gamma\omega} \tag{1}$$

where ω is the angular frequency, $\varepsilon_{\infty} = 1$, the plasma frequency ω_p and damping constant γ are 1.37×10^{16} rad s^{-1} and 4.08×10^{13} rad s^{-1} respectively.

Al_2O_3: The refractive index is given by the experimental results[3].

Ge: In numerical simulation, the data on the MIR refractive index of the Ge are derived from measurements performed by an Ellipsometer working in the MIR (IR-VASE Mark II from J. A. Woollam). This data are plotted in Fig. S1**.**

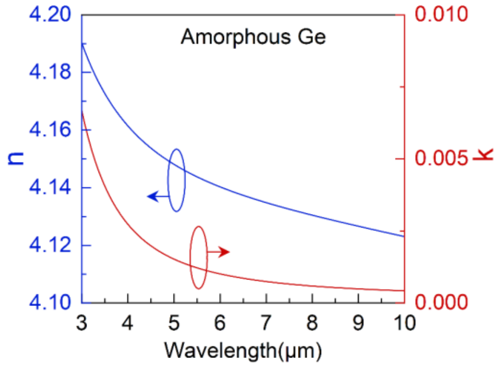

Fig. S1 | The refractive index parameter of Ge. The refractive index of the 600nm Ge thin film measured by an MIR ellipsometer.

Based on the results in the reference[4] and combining the refractive indexes measured by an ellipsometer working at room temperature, we fitted the relationship between the refractive index of Ge and the temperature. The results of the thermo-optic effect of Ge material is shown clearly in Fig. S2(a). Using the fitted results, the output spectra at different temperatures were numerically calculated and presented in Fig. S2(b). It is seen that as the temperature increases, the spectra red shifts. The sensitivity of the resonances shift is quite consistent with the experimental results. This verifies the control of the output wavelength by temperature as a means of adjustment in the experiment.

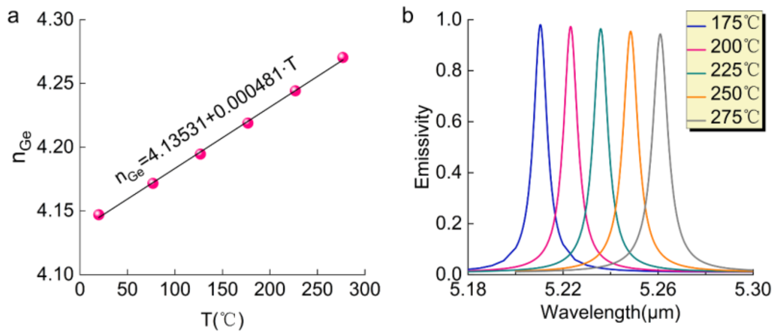

Fig. S2 | The temperature dependence of Ge refractive index. (a) The fitted relationship between the refractive index of Ge and the temperature. (b) Numerically calculated emissivity spectra at different temperatures by using the fitted Ge refractive index.

II. Further numerical calculation results

We characterized the far-field polarization characteristics of all the structures in Fig. 1(c). The polarization states of the Bloch mode can be usually mapped onto the Poincaré sphere[5], whose coordinates are specified by the Stokes parameters S_0, S_1, S_2 and S_3. The *V* point (S_0, S_1, S_2, S_3=0) usually carry integer topological charges at the Γ point. In the continuum, a nondegenerate eigenmode matches only propagating waves with the same in-plane wavevector *k*. The farfield radiation polarization vectors projected onto the *xy* plane is referred to as $d(\boldsymbol{k})=d_x(\boldsymbol{k})\hat{x}+d_y(\boldsymbol{k})\hat{y}$. The characteristic of vortices is their topological charge which is defined as[6]:

$$q=\frac{1}{2\pi}\oint_C d\boldsymbol{k}\cdot\nabla_k\phi(\boldsymbol{k}) \tag{2}$$

where $\phi(\boldsymbol{k})=1/2arg[S_1(\boldsymbol{k})+iS_2(\boldsymbol{k})]$ ($-\pi/2 \le \phi \le \pi/2$) is the orientation angle of the polarization state, and *C* is a simple closed path in the *k*-space. Through numerical calculations, it is found from the results in Fig. S3 that all the structures in Fig. 1(c) except for the final one, exhibit SP-BIC properties at Γ point, with a topological charge of +1. For the final DPS structure, it exhibits *y*-direction polarization characteristics and can radiate towards the far field.

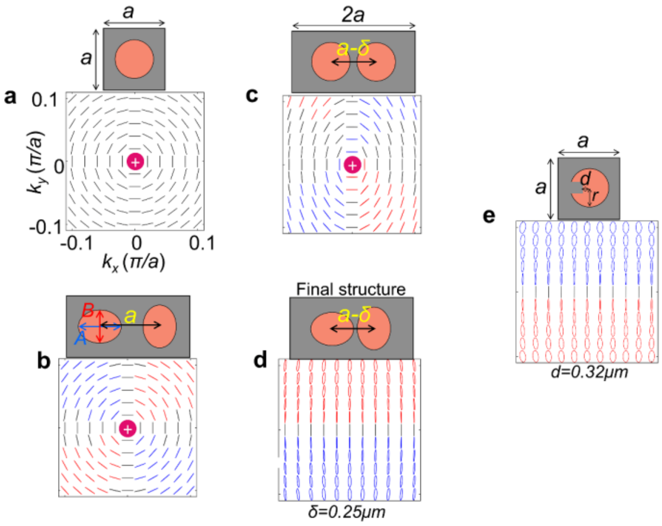

Fig. S3 | The evolution of the far-field polarizations. (a) (b), (c), and (d) represent the far-field polarization of different structures in Fig.1(c). (e) The far field polarization of the QBIC mode in the symmetry breaking SDS.

We numerically investigated the effects of two parameters (*δ* and *h*) on the coupling strength between the two bands in a DPS (Δ=190nm) structure. Fig. S4(a) shows that at *h*=600nm and δ=0nm, there is no coupling between the corresponding eigenmodes of the two bands. When δ is introduced, the two modes couple and form two anti-crossing bands. The coupling strength increases with the increase of *δ*, as shown from Fig. S4(a), (b) to (c). For a fixed value of δ, e.g., 250nm, the coupling strength increases with the increase of *h*, as shown in Figs. S4(d), (b), and(e). When *δ*=250nm and *h*=600nm, the optimal coupling effect is obtained, achieving the appearance of flat dispersion in the high-frequency (blue) band within a large wavevector range.

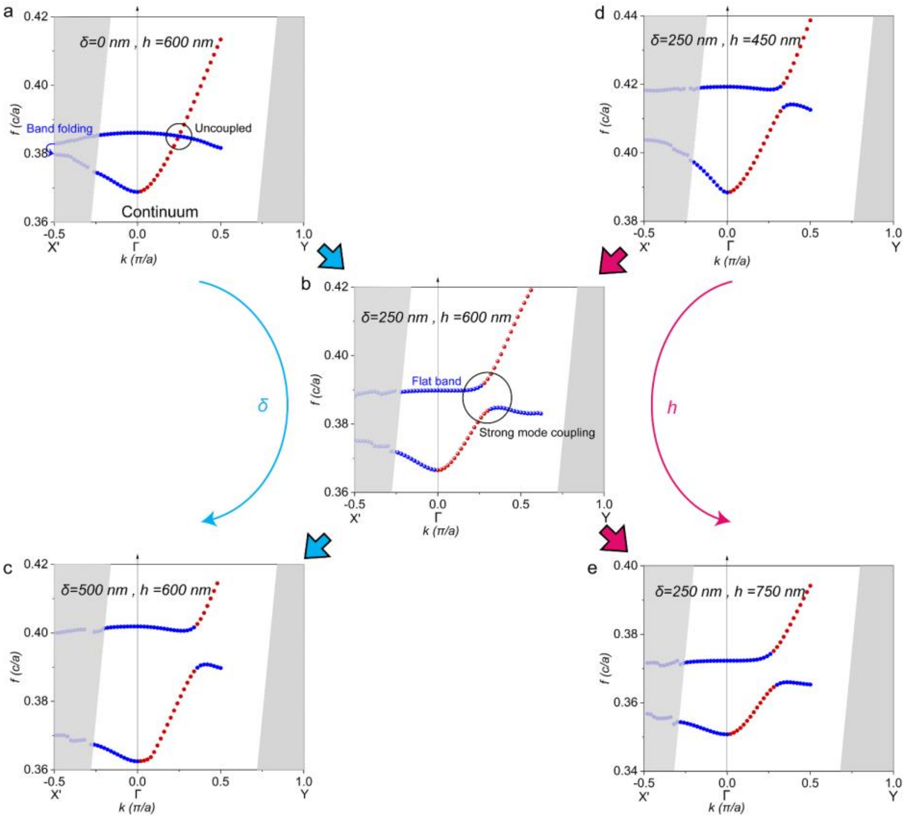

Fig. S4 | The evolution process of the band structure. Changes of the dispersion band under different δ when *h*=600nm(a, b and c) or at fixed δ of 250nm but different structural height *h* (d, b and e). (a) presents the case when there is no coupling. Clearly, as the δ or *h* increases, the coupling strength increases.

For the results of multipole decomposition in this work, based on the following formula[7], in the case of harmonic excitation exp(*iwt*), the sum of the total scattered power can be expressed as:

$$\boldsymbol{I} = \frac{2\omega^4}{3c^3}|\boldsymbol{P}|^2 + \frac{2\omega^4}{3c^3}|\boldsymbol{M}|^2 + \frac{\omega^6}{5c^5}\sum\left|\boldsymbol{Q}^{(e)}_{\alpha\beta}\right|^2 + \frac{\omega^6}{20c^5}\sum\left|\boldsymbol{Q}^{(m)}_{\alpha\beta}\right|^2 + \frac{2\omega^6}{3c^5}|\boldsymbol{T}|^2 \quad (3)$$

where c is the speed of light, ω is the angular frequency, and $\alpha, \beta = x, y, z$. The first and second terms represent the contributions by the electric and magnetic dipoles, respectively, the third and fourth terms from electric and magnetic quadrupoles, respectively, while the fifth term is from toroidal dipole. The moments for the electric dipole (*P*), magnetic dipoles (*M*), toroidal dipole (*T*), electric quadrupole (*QE*), and magnetic quadrupole (*QM*) can be expressed in the Cartesian coordinates as:

$$\boldsymbol{P} = \frac{1}{i\omega}\int \boldsymbol{j} d^3r \quad (4)$$

$$\boldsymbol{M} = \frac{1}{2c}\int (\boldsymbol{r} \times \boldsymbol{j}) d^3r \quad (5)$$

$$\boldsymbol{T} = \frac{1}{10c}\int [(\boldsymbol{r}\cdot\boldsymbol{j})r - 2\boldsymbol{r}^2\boldsymbol{j}] d^3r \quad (6)$$

$$\boldsymbol{Q}^{(e)}_{\alpha\beta} = \frac{1}{i\omega}\int [\boldsymbol{r}_\alpha \boldsymbol{j}_\beta + \boldsymbol{r}_\beta \boldsymbol{j}_\alpha - \frac{2}{3}(\boldsymbol{r}\cdot\boldsymbol{j})] d^3r \quad (7)$$

$$Q^{(m)}_{\alpha\beta} = \frac{1}{3c}\int[(\boldsymbol{r}\cdot\boldsymbol{j})_\alpha \boldsymbol{r}_\beta + (\boldsymbol{r}\cdot\boldsymbol{j})_\beta \boldsymbol{r}_\alpha]d^3r \tag{8}$$

In the DPS structure, the emission spectra under different Al_2O_3 thicknesses (*t*) are shown in Fig. S5 (a), with a slight redshift in the spectra as the thickness increases. Importantly, when *t* is in the range of 300 to 450nm, the emissivity approaches 100%, and the Q factor increases with the increase of *t*, as shown in Fig. S5(b). This is because at larger *t*, the top structure is less affected by the loss of the lower metal. Therefore, in the final structure, *t*=450nm is used to achieve both the high Q and the high peak emissivity, simultaneously.

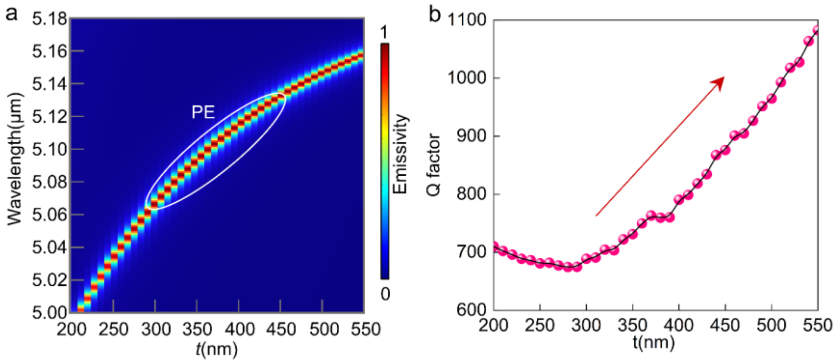

Fig. S5 | The effect of Al_2O_3 thickness on the emission property. (a) Emission spectrum as a function of the Al_2O_3 thickness *t* for the final designed DPS. The white circle marks the area where the emissivity approaches unity. (b) The relationship between the Q factor and the thickness of Al_2O_3.

III. Device fabrication

The full fabrication flow of the sample is shown in Fig. S6. First, a 5 nm thick adhesion layer of Ti, a 100 nm thick Au film and another 3 nm Ti were successively deposited onto a Si substrate using electron beam evaporation(EBE) (DETECH, DE500C) at a deposition rate of 0.5Å/s and the vacuum chamber pressure of 8×10^{-7} Torr. After that, two layers of 450nm Al_2O_3 and 600nm Ge were deposited onto the sample using the same equipment at a deposition rate of 1.5 Å/s. The PMMA resist (950 A4 from KayakuAM) was spin-coated onto the sample at 4000 rpm for 60s, followed by a baking at 180℃ for 90s to obtain a 180 nm thick PMMA layer. The designed structure pattern were exposed onto the PMMA layer by a 50KV electron beam lithography (EBL) system (Raith Voyager). After developing (1:3 MIBK-to-IPA for 35s followed by a 30s rinse in IPA), 20nm Al_2O_3 was evaporated onto the sample and the lift-off process was used to transfer the pattern from PMMA to Al_2O_3 layer. The Al_2O_3 pattern worked as a mask to obtain the Ge structure in a subsequent inductive coupled plasma enhanced reaction ion etching (ICP-RIE) process (Oxford Instruments, Plasma Pro 80 Cobra).

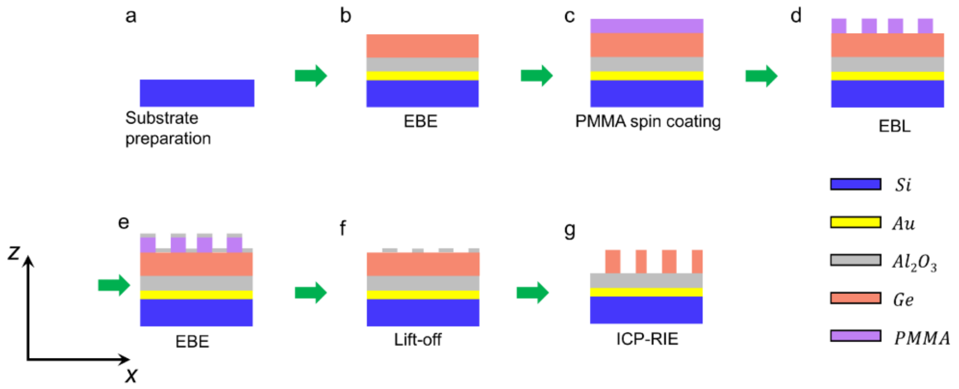

Fig. S6 | Fabrication flow of the sample. (a) Cleaned Si wafer substrates. (b) Au, Al_2O_3 and Ge films were deposited by using the EBE. (c) Spin coating PMMA photoresist. (d) The designed structure pattern were exposed onto the PMMA layer by EBL. (e) The Al_2O_3 was evaporated onto the sample and the lift-off process(f) was used to transfer the pattern from PMMA to Al_2O_3 layer. (g) The Al_2O_3 pattern worked as a mask to obtain the Ge structure in a subsequent ICP-RIE process.

IV. Optical measurements

The emissivity of the fabricated sample was measured by the Fourier transform infrared spectrometer (FTIR) (Bruker Vertex 80-V) equipped with a liquid-nitrogen-cooled mercury cadmium telluride (MCT) detector. A home-made linear polarizer working in the MIR composed of one-dimensional subwavelength array of gold nanowires on a CaF_2 substrate was used for the polarization characterization. The emissivity measurements were carried out by loading the sample onto an emission adapter (Bruker, A540) to heat the sample at different temperatures to induce thermal emissions. Steel plates of the same size blackened by the black soot of candles are used as the standard blackbody reference. Both the blackbody and the fabricated sample were loaded onto the heating plate. When the blackbody and the sample are heated to above 100 ℃, the emitted power was sent to the FTIR system and detected by MCT detector.

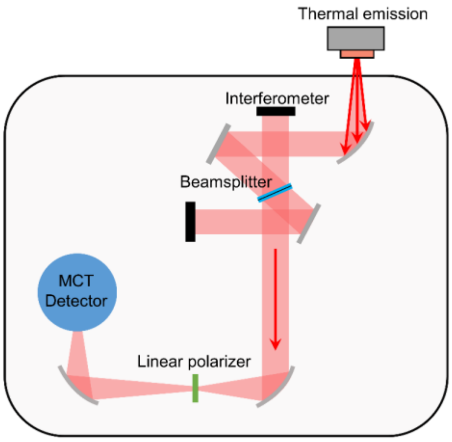

Fig. S7 | Measurement device diagram. The optical path in the FTIR chamber.

To eliminate the thermal noises at high temperatures from the metallic components in the emission adapter, we characterize the normalized emissivity E(λ) from our fabricated thermal emitter using the following equation:

$$E(\lambda) = \frac{e_m(\lambda) - e_{background}(\lambda)}{e_b(\lambda) - e_{background}(\lambda)} \tag{9}$$

where $e_{background}(\lambda)$ is the background emission in the adapter without any samples, $e_m(\lambda)$ and $e_b(\lambda)$ are the measured emissions when the fabricated metasurface structure or the blackbody sample is loaded, respectively.

The morphology of the metasurface sample was characterized by scanning electron microscope (Sigma 500, Zeiss). The data on the refractive index of the Ge in the MIR and its thickness are derived from measurements performed by an Ellipsometer working in the MIR (IR-VASE Mark II from J. A. Woollam).

The schematic of our experimental setup used to characterize the thermal emissions at different output angles are given in Fig. S8.

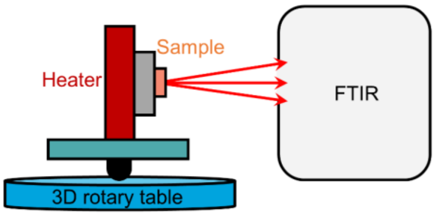

Fig. S8 | Angular resolution measurement. Schematic diagram of simplified experimental apparatus used to characterize the thermal emission at the change of output angle.

V. Further results from the thermal emitter based on the QBIC modes

Fig. S9(a) and (b) present the schematic diagram of the symmetry breaking SDS (SB-SDS). In the absence of perturbation (i.e., without the slot), it corresponds to the schematic diagram in the upper left corner of Fig. 1(b), which exhibits the characteristics of BIC. By introducing the slot into the disk, the structural symmetry is broken and the BIC is transformed into QBIC. For a good comparison, all geometric parameters are assumed to be: a=2 μm, r=1.6 μm, d=0.68 μm, L=0.35 μm, h=0.6 μm, and t=0.45 μm. The dispersion curve in the k_y direction supported by this structure is shown in Fig. S9(c), which is quite consistent with Fig. 2(a) in the main text. When considering all the material losses, the relationship between the Q factor and d were calculated. According to the temporal coupled mode theory(TCMT), PE was achieved when $Q_{abs}=Q_{rad}$, i.e. d=320 nm. At this point, the corresponding Q_{total} factor is 880, which is close to that of the DPS thermal emitter. The far-field emission spectrum is shown in Fig. S9(e). The inset shows the magnetic field amplitude and electric field vector distribution at the center wavelength of 5.055μm, showing the same magnetic dipole mode as SPS and DPS in Fig.2 of the main text. The results from the multipole decomposition analysis in Fig. S9(f) confirms the dominant contribution from the magnetic dipole.

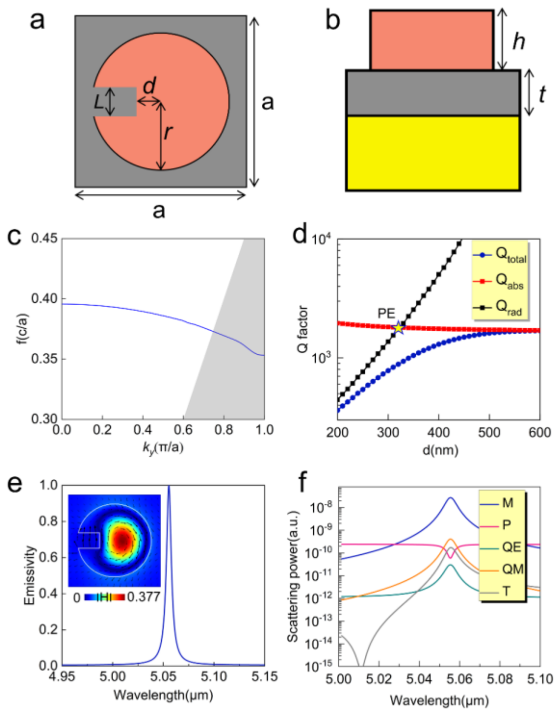

Fig. S9 | Numerical simulation results of SB-SDS. (a) and (b) The top and side schematic views of the SB-SDS, respectively. (c) The QBIC band supported by the structure in (a). The gray area represents the guided mode area below the light line. (d) The relationship between Q_{total}, Q_{abs}, and Q_{rad} as a function of *d*. The blue, red and black curves represent Q_{total}, Q_{abs}, and Q_{rad}, respectively. PE is achieved when Q_{abs}=Q_{rad}, as shown by the yellow star. This corresponds to *d*=320nm which is used as the final SB-SDS thermal emitter parameter. (e) the emission spectrum and (f) multipole decomposition of the resonance in the normal output direction of the thermal emitter, respectively. The inset in (e) shows the amplitude of the magnetic field and the vector distribution of the electric field at the peak wavelength.

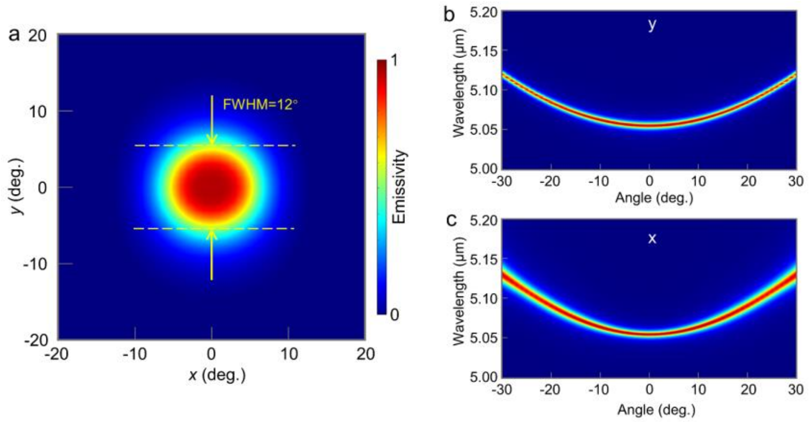

Fig. S10 | The spatial output characteristics of the SB-SDS. (a)The emissivity as a function of the output angle in 3D space, calculated at the resonance wavelength of the SB-SDS thermal emitter in the normal direction. (b) and (c) The results of a 2D mapping of the emissivity as a function of the wavelength and the output angle in either the *y* or *x* direction, respectively.

The output emissivity of the SB-SDS towards different angles in the 3D space at the same wavelength of 5.055 μm is shown in Fig. S10(a). The FWHM is 12°, indicating a faster decrease of the emissivity at increasing output angles, compared to our proposed rainbow-free thermal emitter in the main text. The 2D mapping of the emissivity as a function of the wavelength and the output angle in either the *y* or *x* direction are presented in Figs. S10(b) and (c), respectively. It can be seen that the resonance wavelength has a strong dependence on the output angle. In other words, the emission from the SB-SDS exhibits a significant rainbow effect.

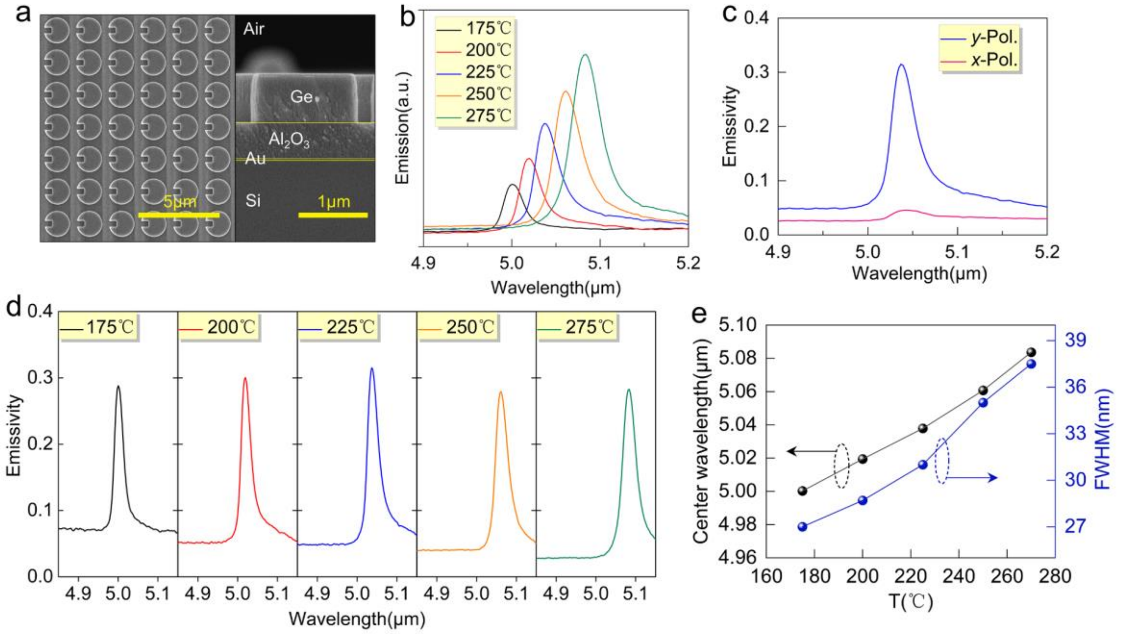

Fig. S11 | Experimental results of SB-SDS thermal emitters. (a) Top and *xz* cross-section views of the fabricated SB-SDS obtained with SEM. (b) Emission intensity of the fabricated samples at different temperatures (175, 200, 225, 250, and 275 ℃) under *y*-polarization. (c) Normalized emissivity spectra under different polarizations at 225 ℃. (d) Normalized emissivity spectra at five different temperatures. (e) Extracted center wavelength and linewidths from (d).

Fig. S11(a) presents top- and side- scanning electron microscope (SEM) images of the device. We employed the same heating adapter from Bruker to heat the sample to high temperatures and used FTIR to characterize the thermal emission from the device. The measured thermal emission spectra under *y* polarization and at different temperatures (175, 200, 225, 250, and 275℃) are shown in Fig. S11(b), exhibiting increasing emission intensity with temperatures. The polarization output characteristics of the device were verified by rotating the polarizer, and shown in Fig. S11(c). By normalizing the emission from the fabricated sample to that from a blackbody emitter (a candle-smoked black steel plate with the same area size as the sample) heated to the same temperature, the emissivity spectrum can be obtained and shown in Fig. S11(d). Compared to DPS thermal emitters, the emissivity is evidently reduced. This is partly attributed to a larger mismatch between Q_{rad} and Q_{abs}, which leads to a further deviation from the critical coupling condition. On the other hand, the steep dispersion leads to the superimposing of different output wavelengths from multiple angles, causing the power to be averaged. We extracted the center wavelength and the linewidth of the emission peak at different temperatures from the results in Fig. S11(d). As shown in Fig. S11(e), similar spectral shift as a function of the temperature is found for the SB-SDS structure as in our DPS emitter. So it is mainly the result of the

thermo-optic effect of Ge. The increasing linewidth at higher temperatures, compared to the results in Fig. 4(e), is mainly attributed to an increase in the output power at larger wavelengths due to the large collection angle provided by the entrance window of the FTIR. This can be seen from the increasing resonance asymmetry at larger temperatures shown in Fig. 4(d).

Fig. S12 shows the dependence of emissivity and polarization at the central emission wavelength from the SB-SDS based on the QBIC modes. The results indicate that the output characteristics of this thermal emitter is linearly polarized in the *y*-direction, while the emissivity is 0 under *x*-polarization.

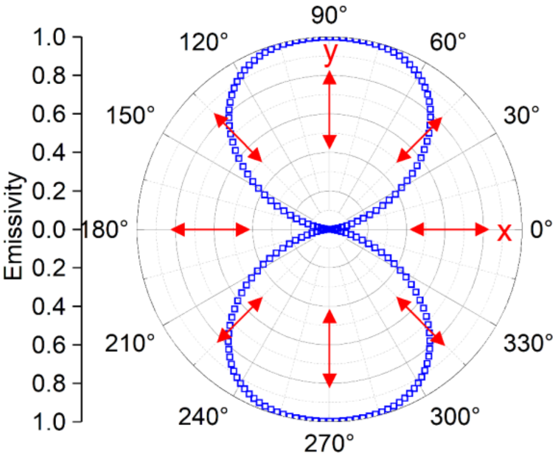

Fig.S12| Polarization characteristics. Polarization dependence of peak wavelength emission intensity of SB-SDS.

Fig. S13 shows the Q-factors extracted from Figs.5 (b), (c), (e), and (f). For DDS that supports flat band, its Q-factor remains almost unchanged as the wave vector increases. Due to the simultaneous collection of multi-angle thermal emissions by the FTIR system, for SDS that supports rainbow bands, it is seen that the Q-factor rapidly decreases with the increase of output angle.

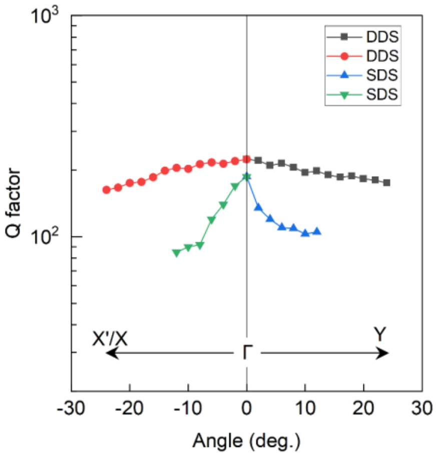

Fig. S13 | Q-factors in our experiments. The Q-factors of the two structures extracted from Figs.5 (b), (c), (e), and (f) at different output angles. The black and blue curves represent the results in the ΓY direction, the red curve represents the results in the ΓX direction, and the green curve represents the results in the ΓX' direction.

VI. Comparion of the output power between our rainbow-free and regular thermal emitters

We compare the collected level of powers of narrow-band thermal emissions from two different types of thermal emitters. One is the regular type of thermal emitter with rainbow effect based on the QBIC or QGMs, and the other is the rainbow-free thermal emitter proposed in this work. For the former case, a spatial filter like a hole in an opaque screen is required to select the spectral component. Due to the steep dispersion of the QBIC or QGMs, the resonance frequency is highly dependent on the output angle. As a result, the hole should be pretty small to ensure the final output with an adequately narrow band. For the latter case, a focusing lens can be used to collect the radiations within a large angle (We keep the angle to be 17.5 ° based on the results in the main text). For a fair comparison, the size of the emitter in the first case is assumed to have the same area with the size of the lens in the second case. The two schemes with the respective output characteristics are shown in Fig. S14.

One can obtain the emitted power from the first case as follows:

$$P_1 = \frac{\Delta\theta}{180^{\circ}} \eta\pi(0.5D_1)^2 \tag{10}$$

where $\Delta\theta$ is the full-width at half maximum in the angular distribution of the thermal emissions at a specific wavelength, η is the coefficient of thermal emissions from unit area size. From the results in our previous studies[8,9], it is known that $\Delta\theta$ is very small and its value is dependent on the objective emission bandwidth and which part of the steep dispersion is used. For the thermal emission with the bandwidth at the order of 10nm, a value of 0.43 ° is used for rough estimation based on our previous results[9].

For the second case, the collected power is:

$$P_2 = \eta S_{eff} = \eta\pi(0.5D_1 - L_0 tan(17.5°))^2 \tag{11}$$

where S_{eff} is the effective area from which the thermal radiations can be collected within the angle of 17.5 °, to ensure that the flat band effect works. L_0 is the distance between the focusing lens and the sample. One can see that the collected power in the second case is dependent on the value of L_0. To make the estimations practical, we assume that L_0=0.5*D_1, both can be assumed at the length scale of millimeters.

With above assumptions, one can calculate that the power ratio from the two cases:

$$\frac{P_2}{P_1} = \frac{(1-tan(17.5°))^2}{180} = 168 \tag{12}$$

This estimation result suggests that our scheme of combining the rainbow-free thermal emitter with a focusing lens can increase the power output by two orders of magnitude compared to the case of using large-area rainbow-type thermal emitters with a spatial filter.

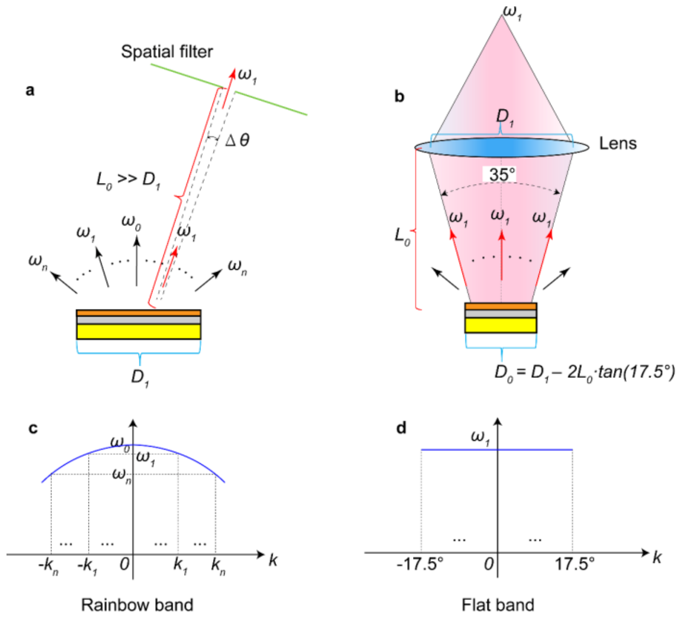

Fig. S14 | Comparison of the emission power from two structures with different dispersion characteristics. (a) and (b) Two schemes of obtaining large-power thermal emissions with high temporal coherence. (c) and (d) The spectral properties of the two types of thermal emitters at a function of wavevector.

VII. Emisson properties at the presence of fabrication errors

Fig. S15 presents the calculated influence of fabrication errors on the emission spectrum. A schematic diagram of the structure with an increase or decrease of 20 nm in the long/short axis of the elliptical disks is shown in Fig. S15(a). The calculated spectral shifts under these two variations in (a) are presented in Fig. S15(b). For the fabrication error of 20 nm in either dimension, it can be seen that a spectral shift of approximately 0.04 μm can be observed away from the objective target wavelength of 5.131 μm. It is seen from the results in Fig. 4(d) that the center wavelength of the emission spectrum from our experimentally fabricated structure is 5.112 μm at 175 ℃ and 5.163 μm at 250 ℃. This shows that the spectral shift due to fabrication errors can be easily compensated by the change of temperature. In addition, through calculations, it can be seen that the dispersion group velocity and Q-factor remain almost unchanged under an error of 20 nm. This indicates that the structure has a high tolerance on the fabrication errors.

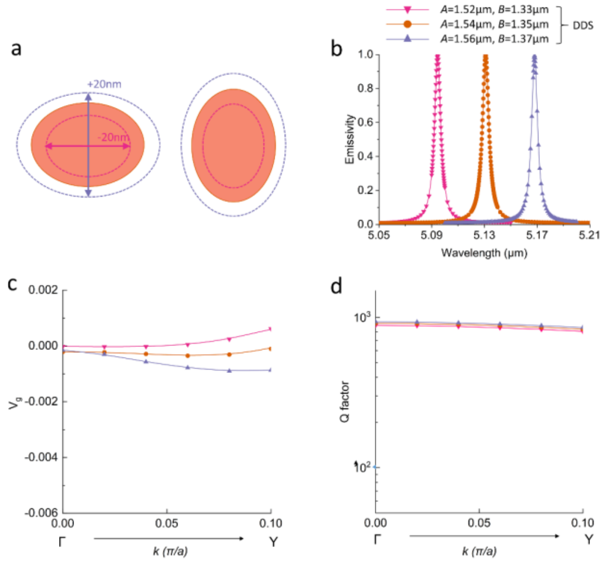

Fig. S15 | Emisson properties at the presence of fabrication errors. (a) A schematic diagram of a structure with an increase or decrease of 20 nm on the long/short axis. (b) The emission spectra of structures with different sizes, where the orange line represents the emission spectra of the original design structure size, the red line represents the emission spectra of structures with a reduction of 20 nm, and the blue line represents the emission spectra of structures with an increase of 20 nm. (c) The calculation results of dispersion group velocities supported by the structures in (a). (d) The dependences of Q-factor on the wavevector for three different structures with 20 nm fabrication errors are shown.

VIII. Discussions on the emission results from finite and infinite sized metasurfaces

Fig. S16 provides schematically a comparison between the performances of finite and infinite size metasurfaces as thermal emitters. Both finite and infinite structures supporting rainbow bands exhibit broadband emission responses. For finite sized metasurfaces supporting this kind of dispersion band, the truncation of sample is related with more wavenumbers, which leads to an excitation of multiple resonances. The overlap of them will deteriorate the Q-factor, giving rise to a degradation of the temporal coherence. For flat band emitters, the requirement on the metasurface size is less demanding, considering that the flat-band is always associated with the slow-light effect.

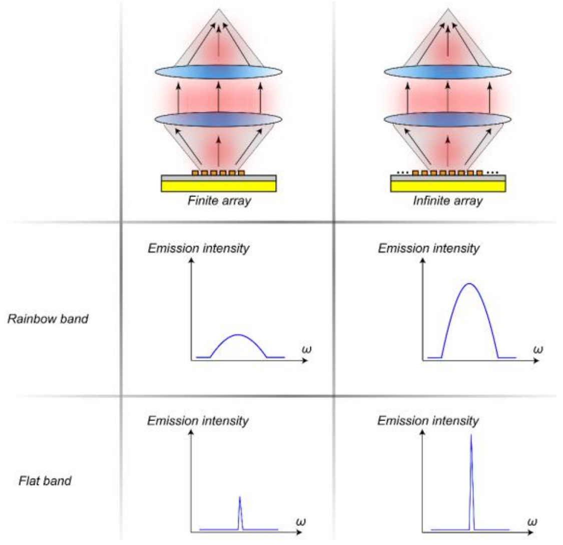

Fig.S16 | Comparison diagram of emission performance between finite and infinite size metasurface structures. Both finite and infinite structures supporting rainbow bands exhibit broadband emission responses. For flat band emitters, the requirement on the metasurface size is less demanding, considering that the flat-band is always associated with the slow-light effect.

References

1. Ordal, M. a, Bell, R. J., Alexander, R. W., Long, L. L. & Querry, M. R. Optical properties of fourteen metals in the infrared and far infrared: Al, Co, Cu, Au, Fe, Pb, Mo, Ni, Pd, Pt, Ag, Ti, V, and W. *Appl. Opt.* **24**, 4493 (1985).
2. Liu, N., Mesch, M., Weiss, T., Hentschel, M. & Giessen, H. Infrared perfect absorber and its application as plasmonic sensor. *Nano Lett.* **10**, 2342–2348 (2010).
3. Palik.E.D. Handbook of Optical Constants of Solids. *New York Acad.* 815–835 (1985).
4. Li, H. H. Refractive index of silicon and germanium and its wavelength and temperature derivatives. *J. Phys. Chem. Ref. Data* **9**, 561–658 (1980).
5. Liu, W. *et al.* Circularly polarized states spawning from bound states in the continuum. *Phys. Rev. Lett.* **123**, 116104 (2019).
6. Zhen, B., Hsu, C. W., Lu, L., Stone, A. D. & Soljačić, M. Topological nature of optical bound states in the continuum. *Phys. Rev. Lett.* **113**, 257401–5 (2014).
7. Yang, L., Yu, S., Li, H. & Zhao, T. Multiple Fano resonances excitation on all-dielectric nanohole arrays metasurfaces. *Opt. Express* **29**, 14905 (2021).
8. Sun, K., Sun, M., Ma, Y., Shi, Y. & Han, Z. Ultra-narrow bandwidth mid-infrared thermal emitters achieved with all-dielectric metasurfaces. *Int. Commun. Heat Mass Transf.* **143**, 106728 (2023).
9. Sun, K., Levy, U. & Han, Z. Thermal Emission with High Temporal and Spatial Coherence by Harnessing Quasiguided Modes. *Phys. Rev. Appl.* **20**, 024033 (2023).
